# Supplementary material for: Oral Exposure to Titanium Dioxide E171 and Zinc Oxide Nanoparticles Induces Multi-Organ Damage in Rats: Role of Ceramide
Source: Int J Mol Sci. 2024 May 28;25(11):5881. doi: 10.3390/ijms25115881 (PMC11172338; doi:10.3390/ijms25115881)
Supplement: Supplementary file 1 [file ijms-25-05881-s001.zip › ijms-2981612-supplementary.pdf]

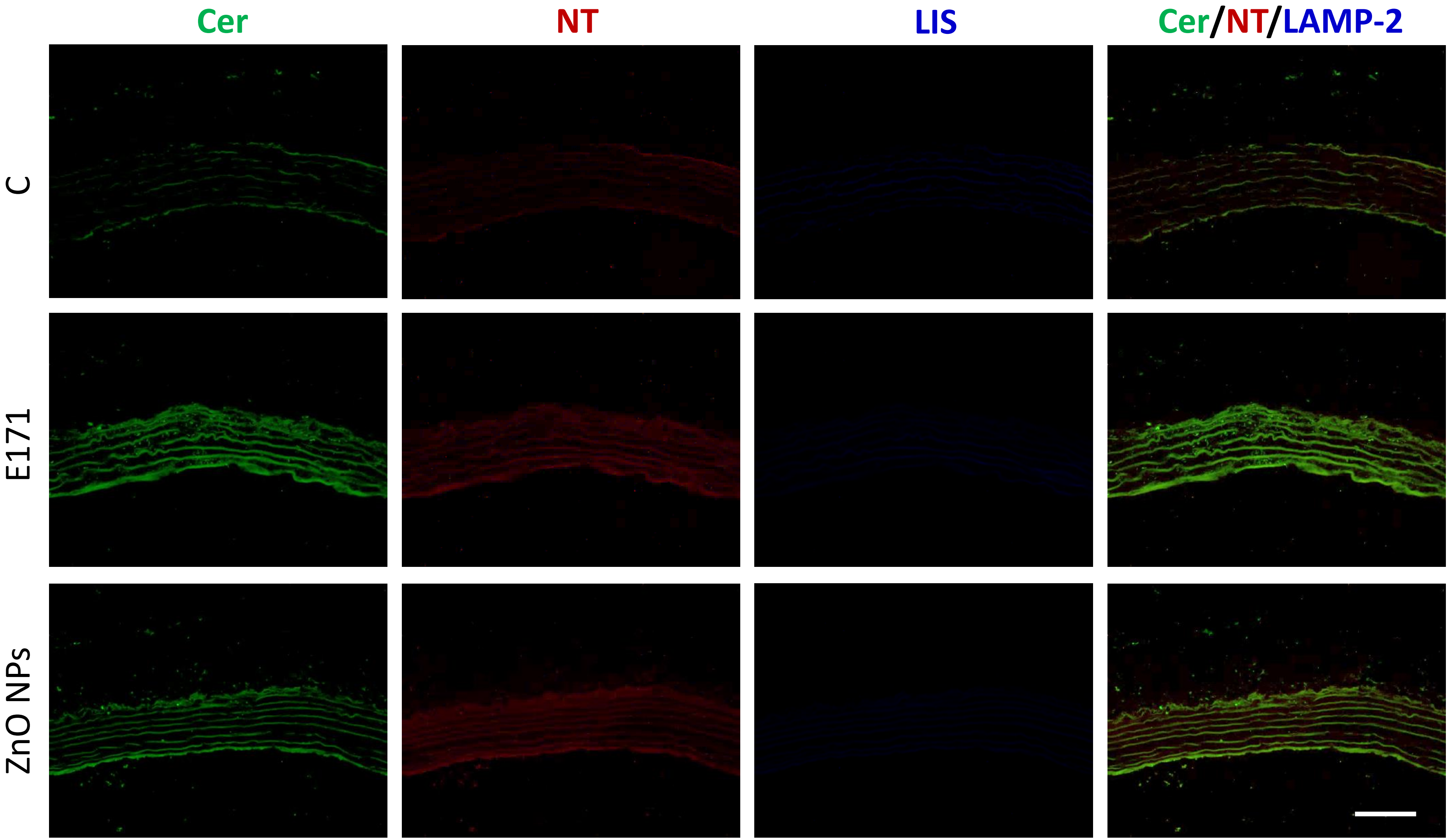


Figure S1. Representative images of ceramide (Cer), nitrotyrosine (NT) and LAMP-2 immunodetection in dorsal aorta of control (C) rats and rats treated with E171 and ZnO NPs. Bar=100µm.

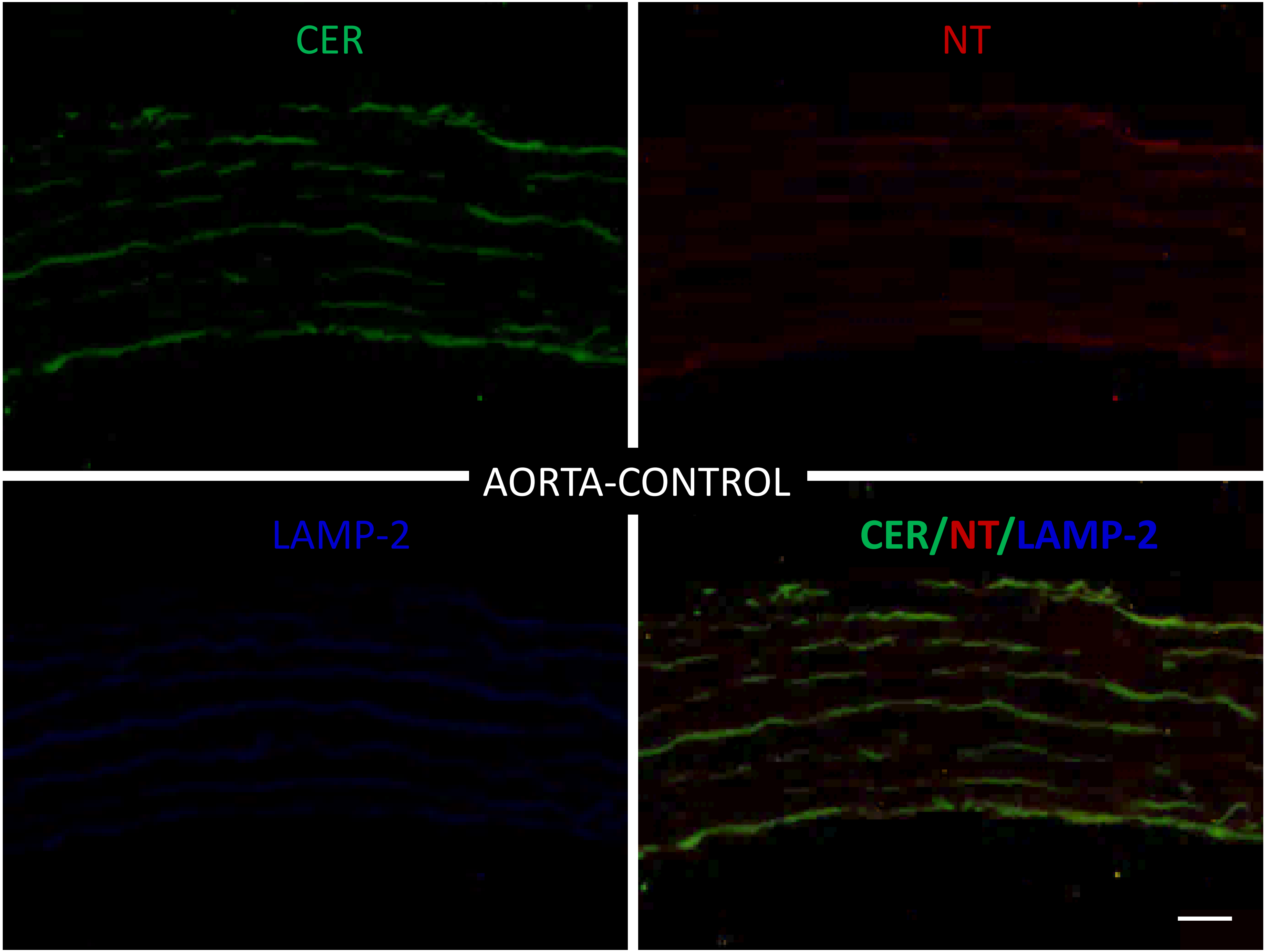
CER
NT
AORTA-CONTROL
LAMP-2
CER/NT/LAMP-2

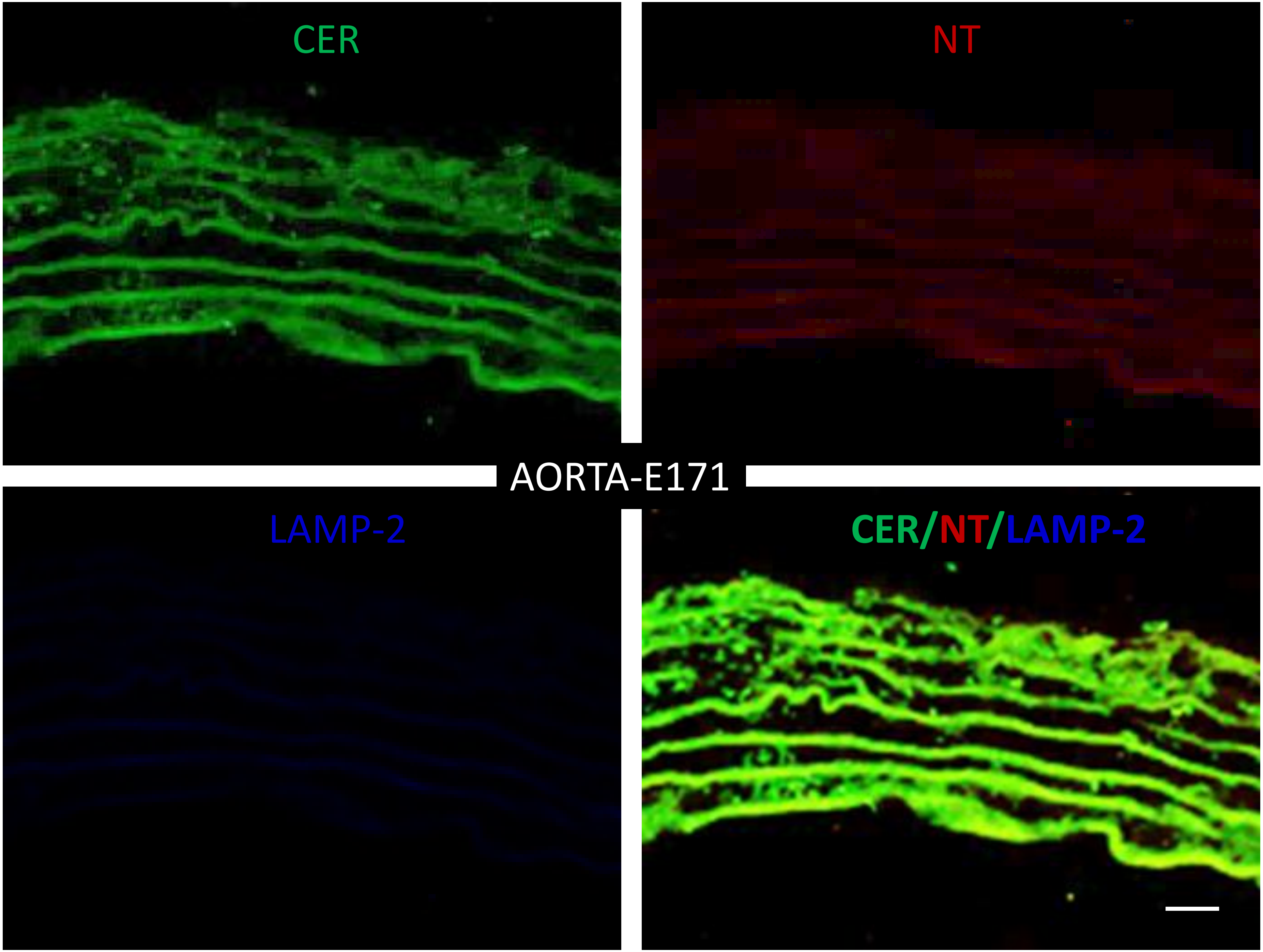
CER
NT
AORTA-E171
LAMP-2
CER/NT/LAMP-2

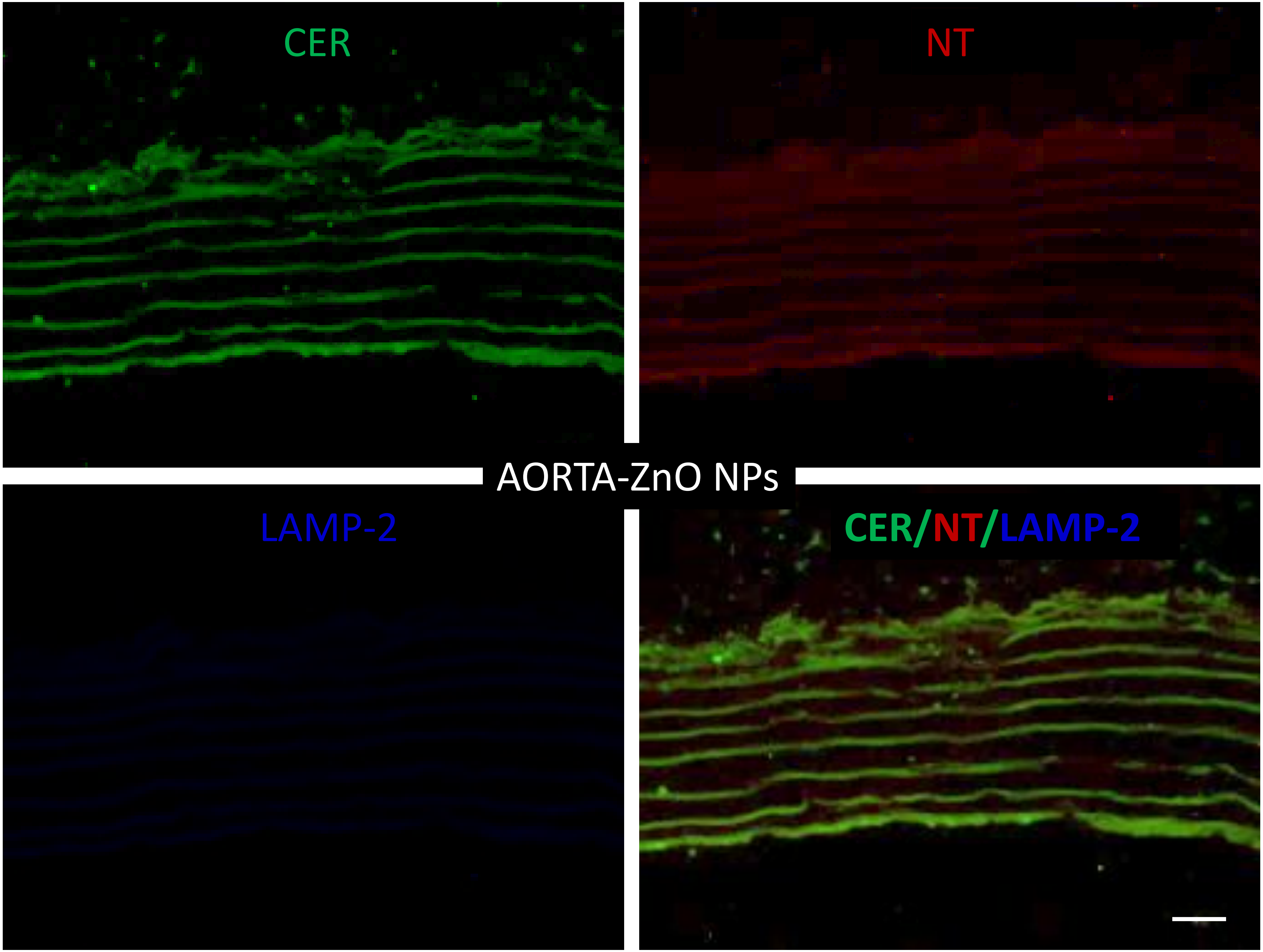
CER
NT
AORTA-ZnO NPs
LAMP-2
CER/NT/LAMP-2

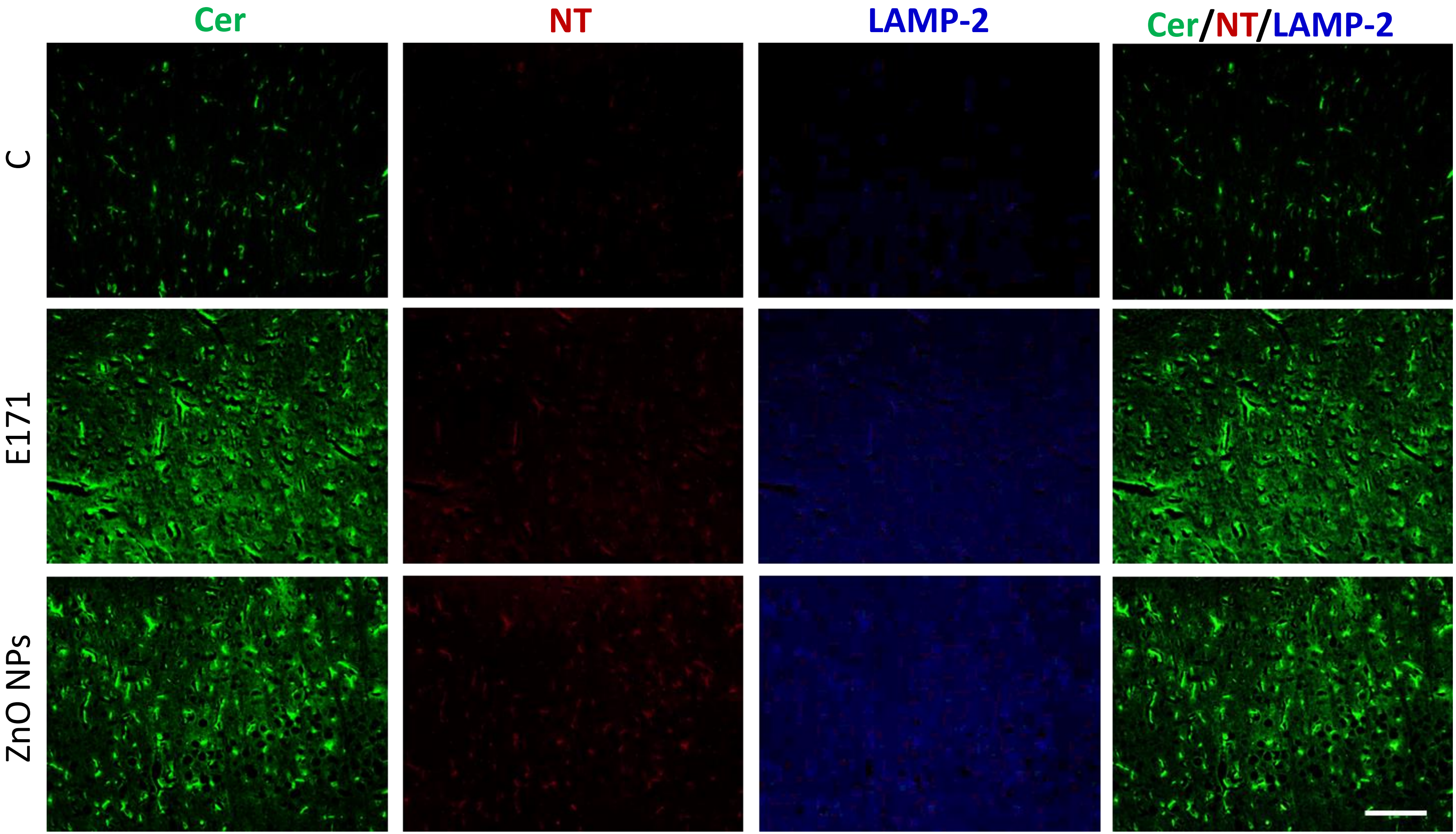


Figure S2. Representative images of ceramide (Cer), nitrotyrosine (NT) and LAMP-2 immunodetection in brain cortex of control (C) rats and rats treated with E171 and ZnO NPs. Bar=100µm.

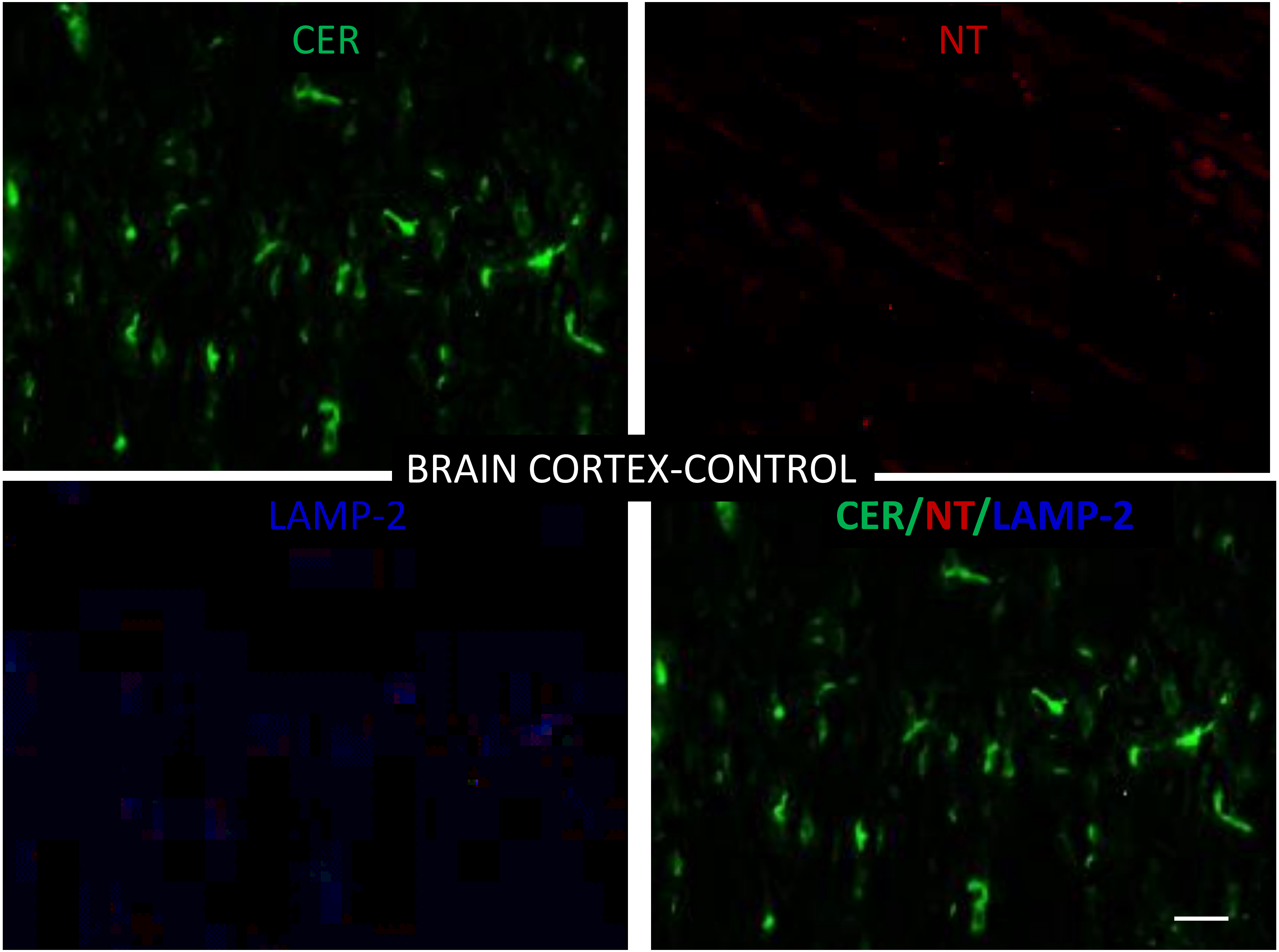
CER
NT
BRAIN CORTEX-CONTROL
LAMP-2
CER/NT/LAMP-2

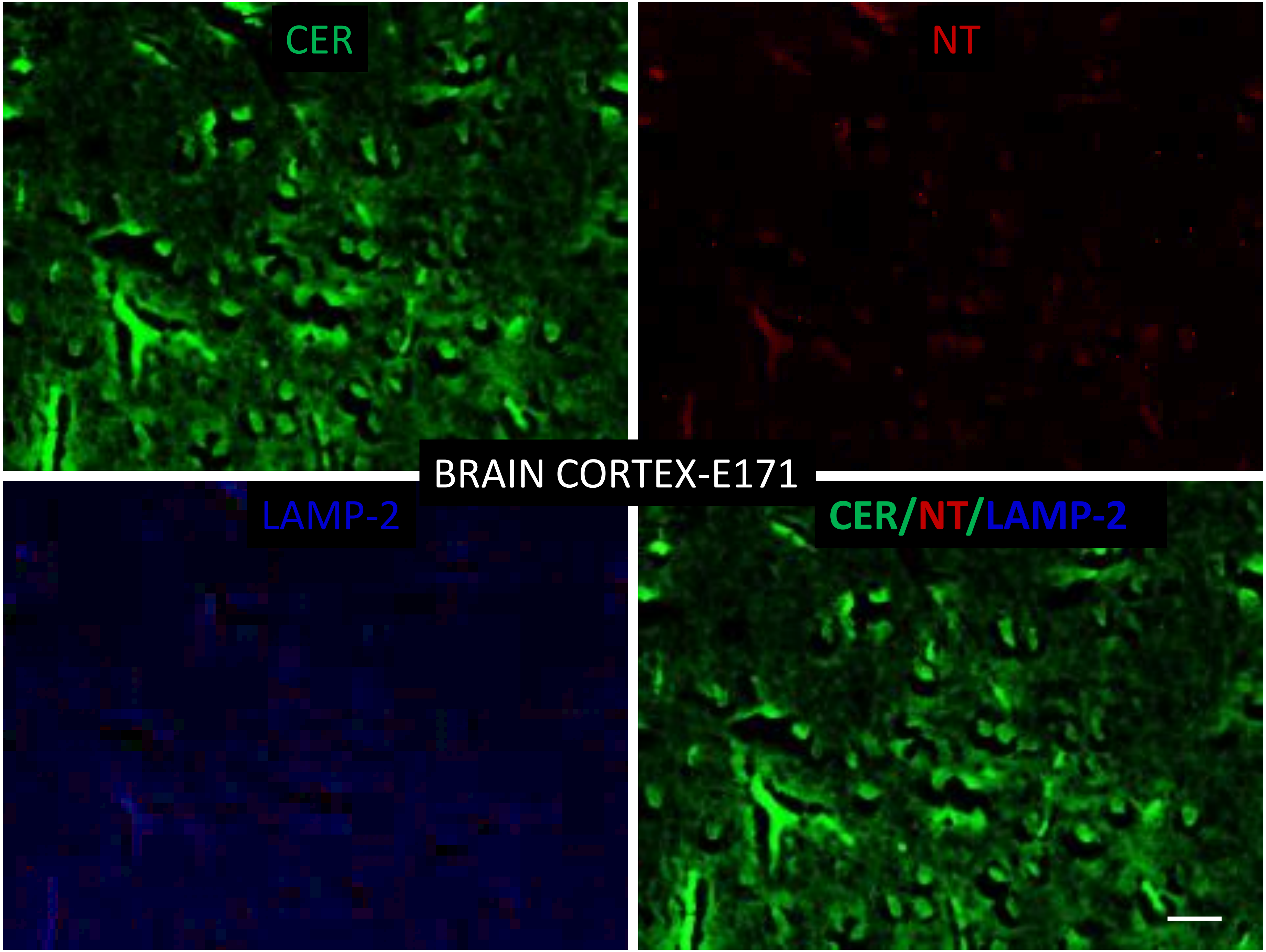
CER
NT
BRAIN CORTEX-E171
LAMP-2
CER/NT/LAMP-2

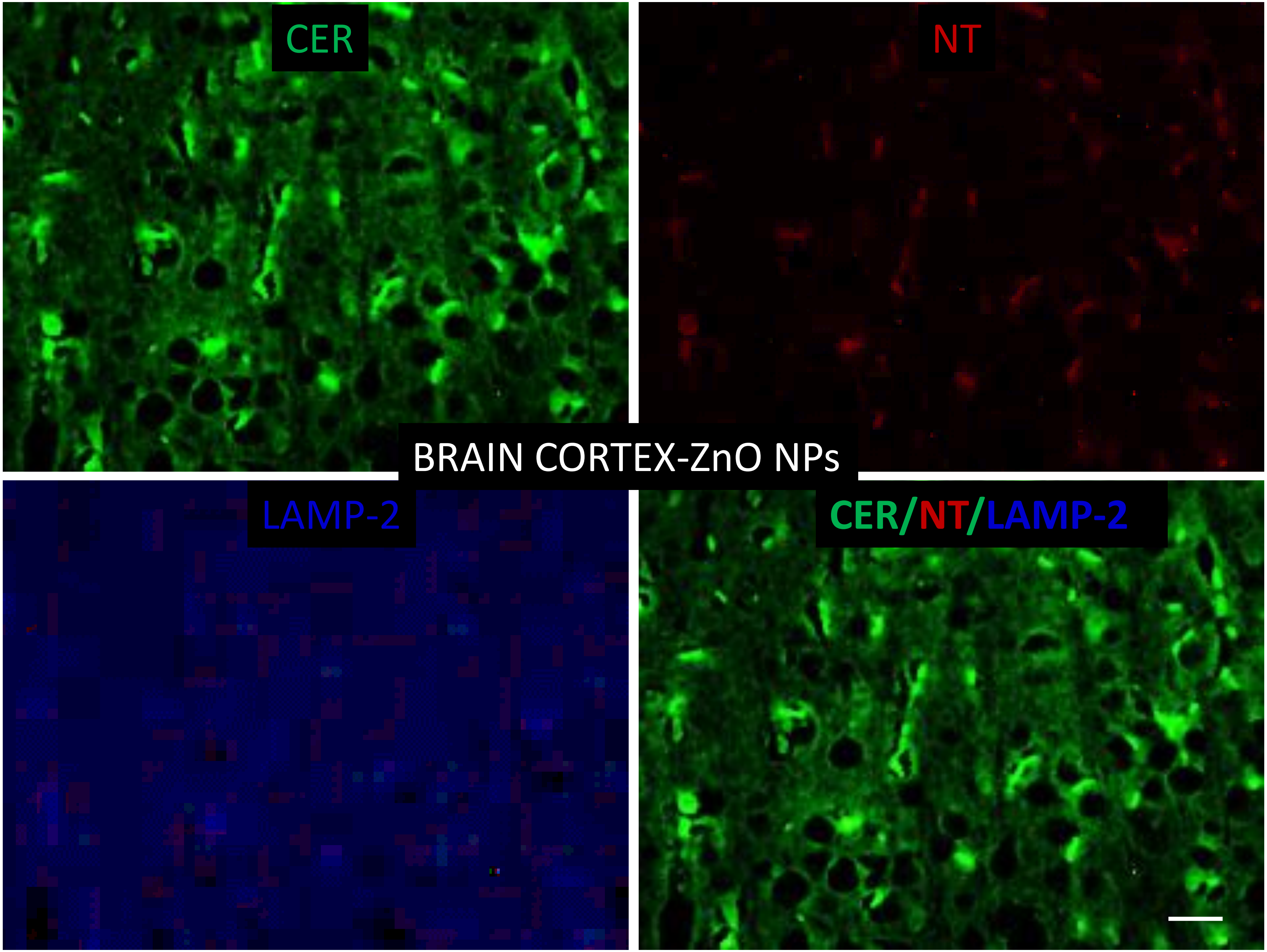
CER
NT
BRAIN CORTEX-ZnO NPs
LAMP-2
CER/NT/LAMP-2

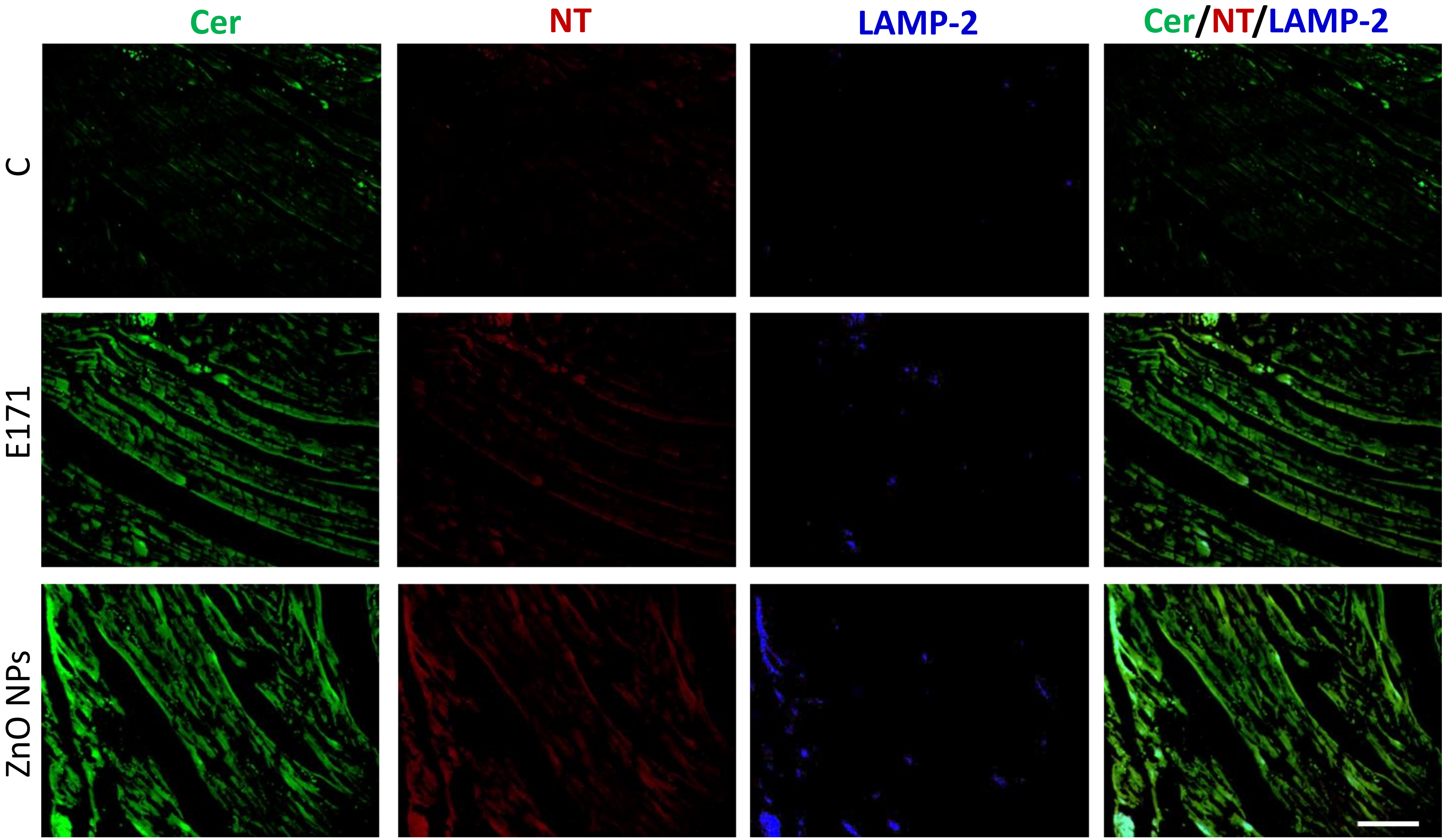


Figure S3. Representative images of ceramide (Cer), nitrotyrosine (NT) and LAMP-2 immunodetection in heart of control (C) rats and rats treated with E171 and ZnO NPs. Bar=100µm.

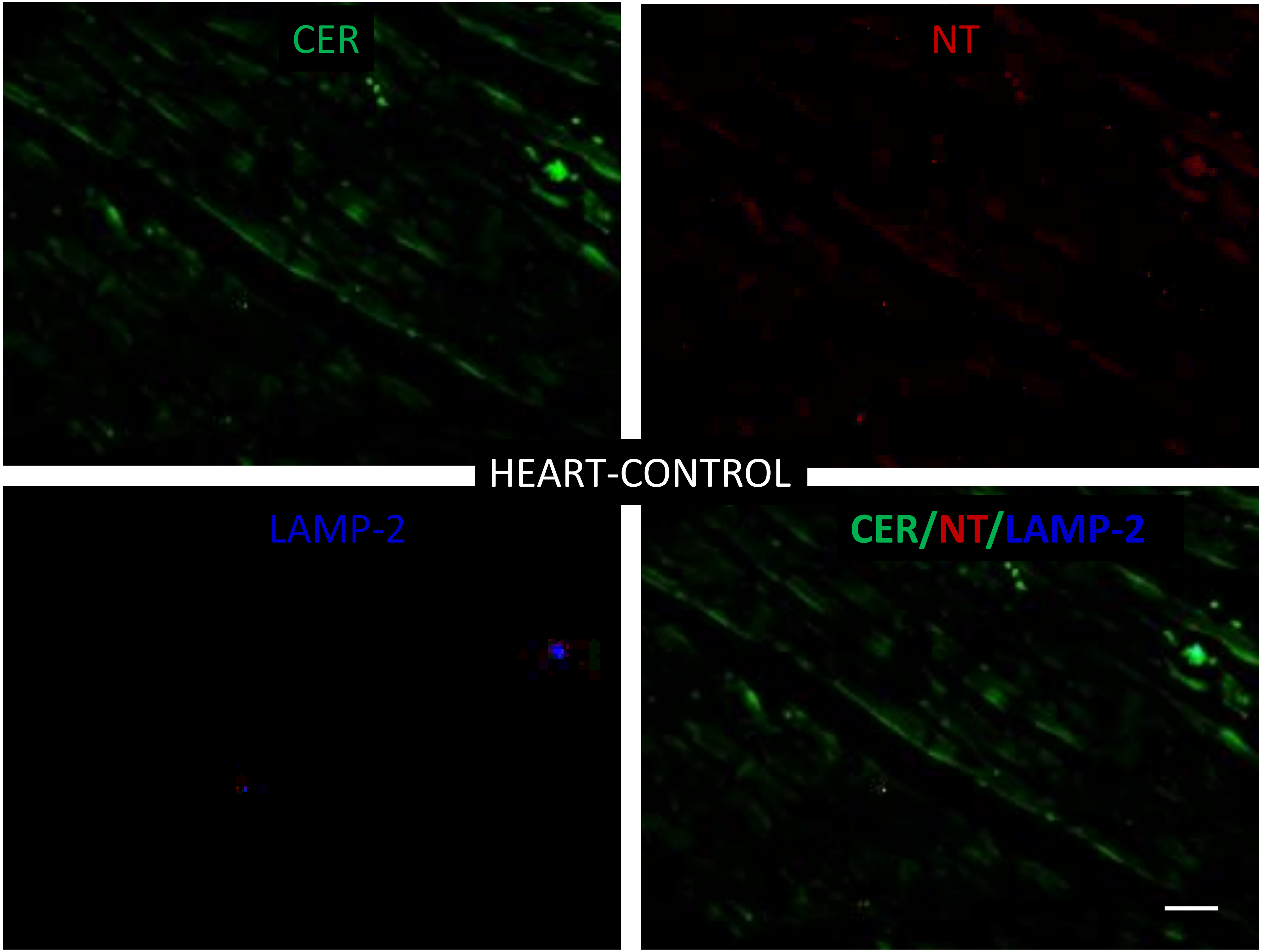
CER
NT
HEART-CONTROL
LAMP-2
CER/NT/LAMP-2

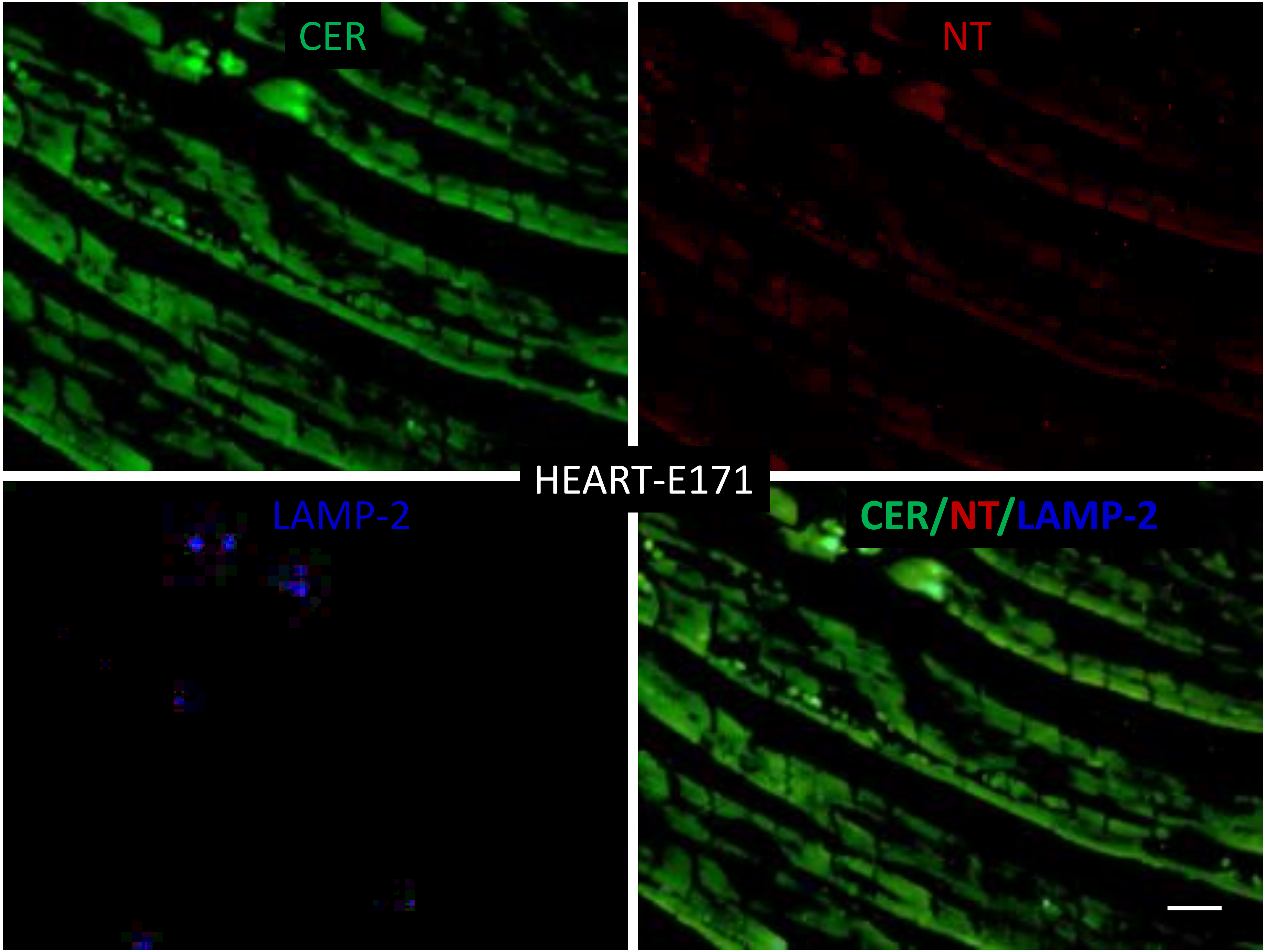
CER
NT
HEART-E171
LAMP-2
CER/NT/LAMP-2

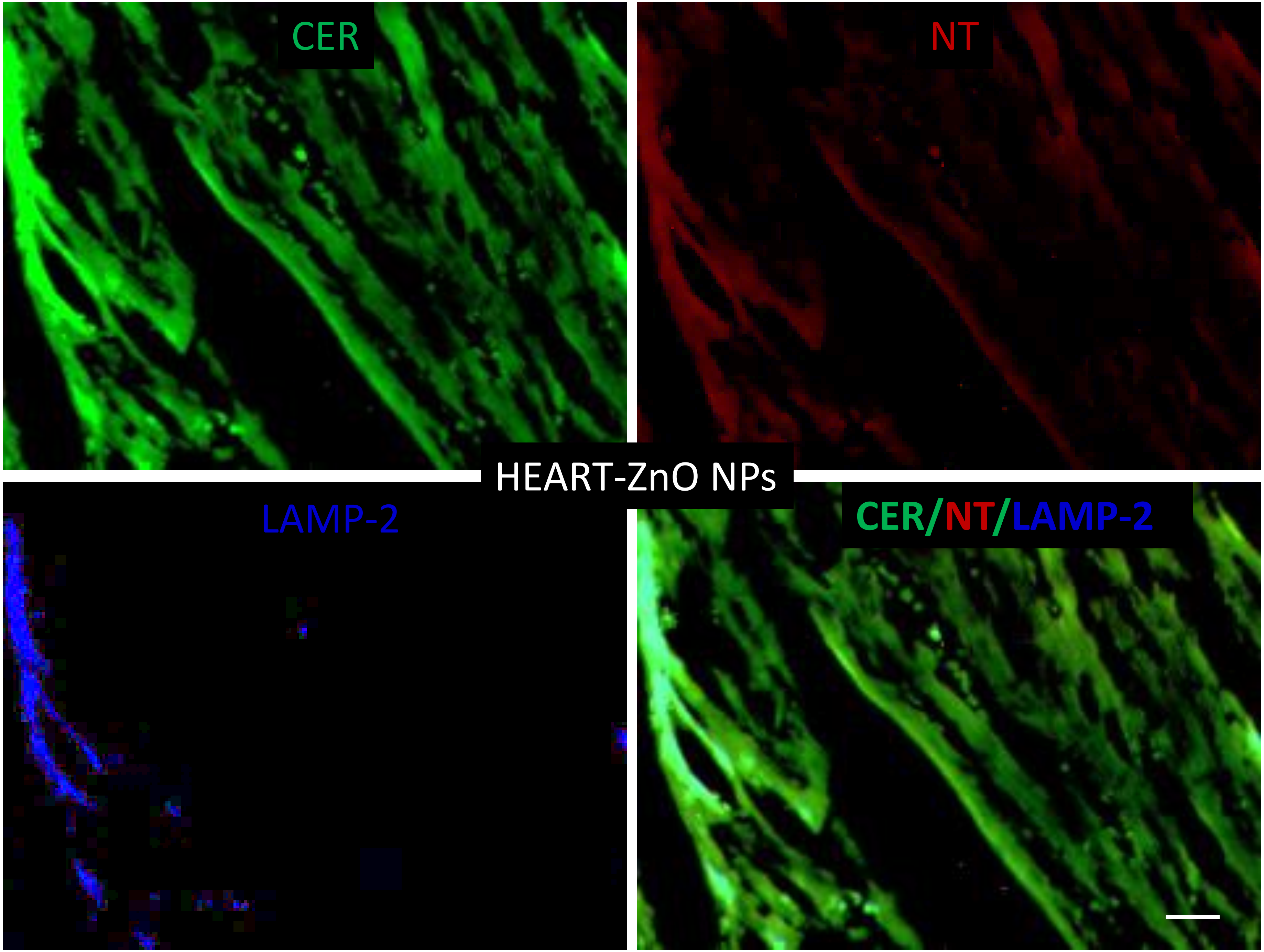
CER
NT
HEART-ZnO NPs
LAMP-2
CER/NT/LAMP-2

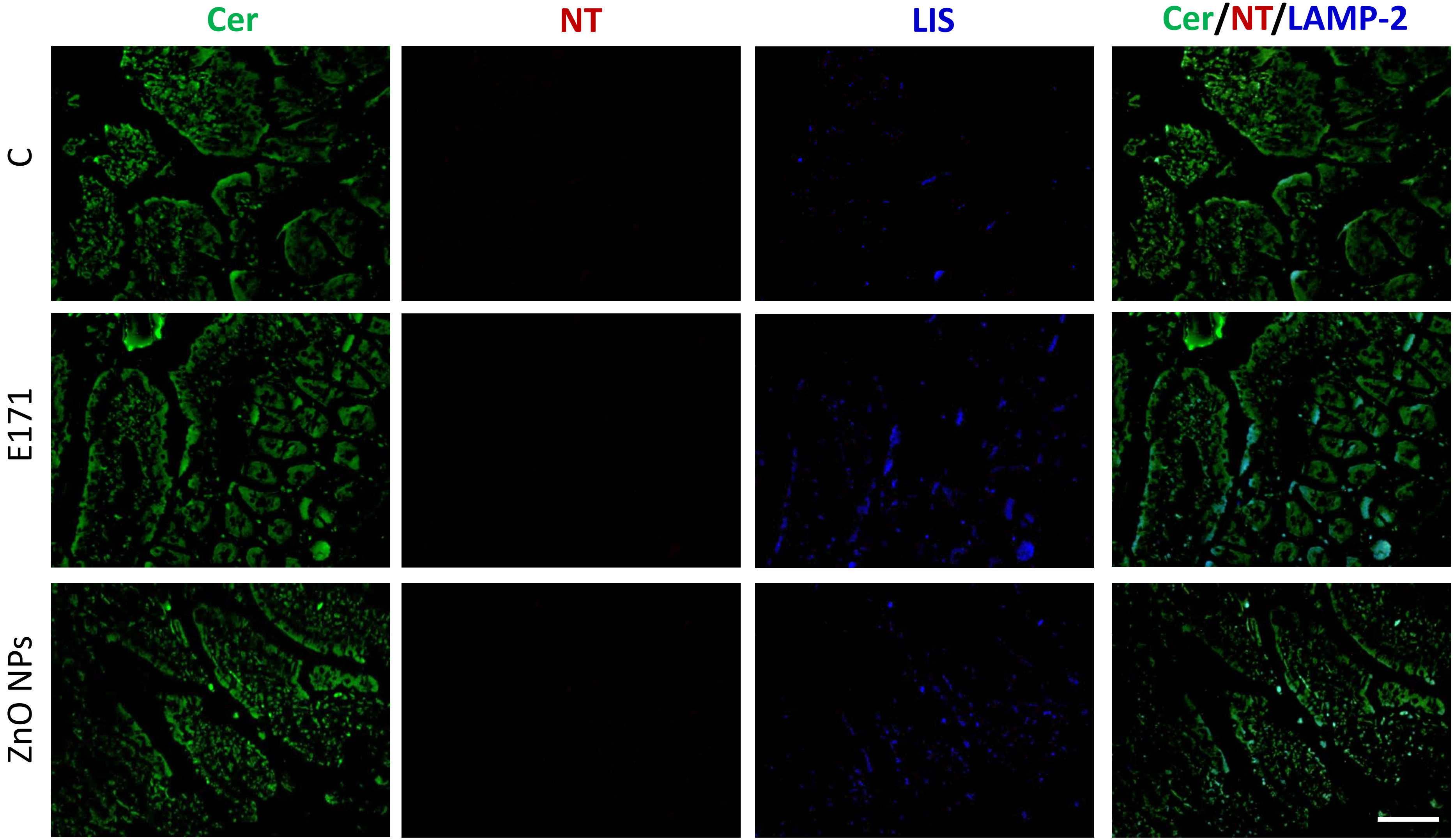


Figure S4. Representative images of ceramide (Cer), nitrotyrosine (NT) and LAMP-2 immunodetection in small intestine of control (C) rats and rats treated with E171 and ZnO NPs. Bar=100µm.

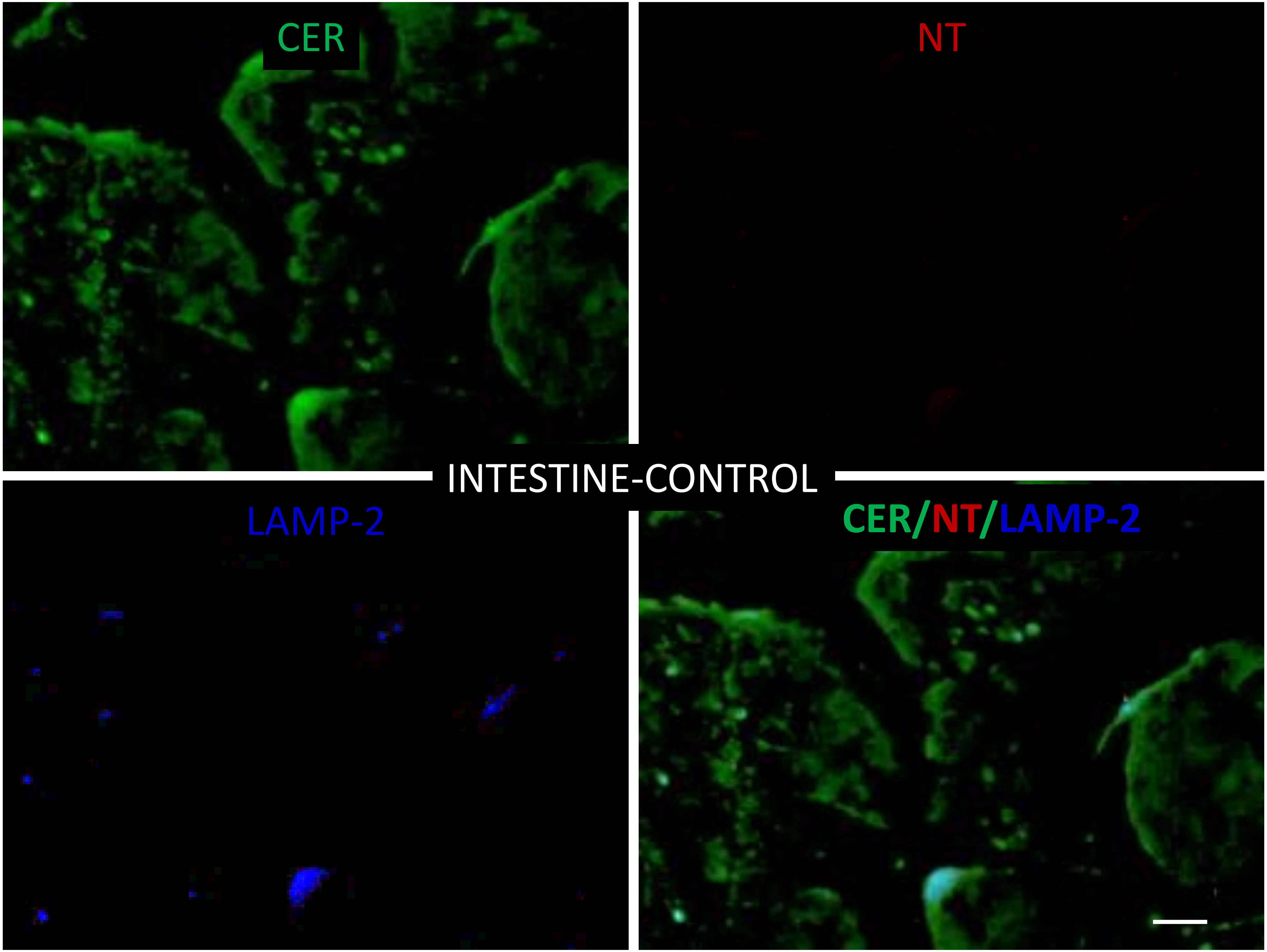
CER
NT
INTESTINE-CONTROL
LAMP-2
CER/NT/LAMP-2

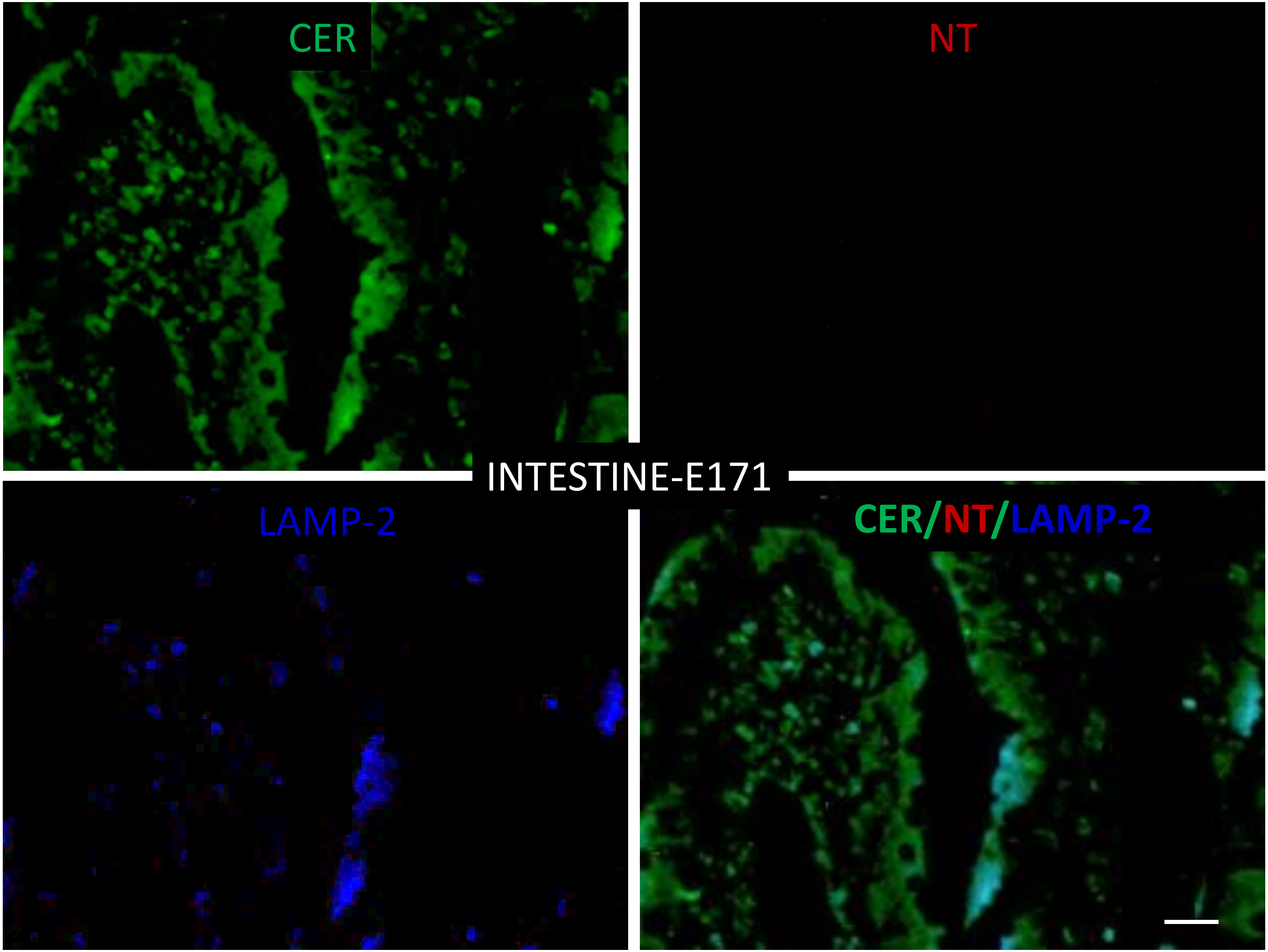
CER
NT
INTESTINE-E171
LAMP-2
CER/NT/LAMP-2

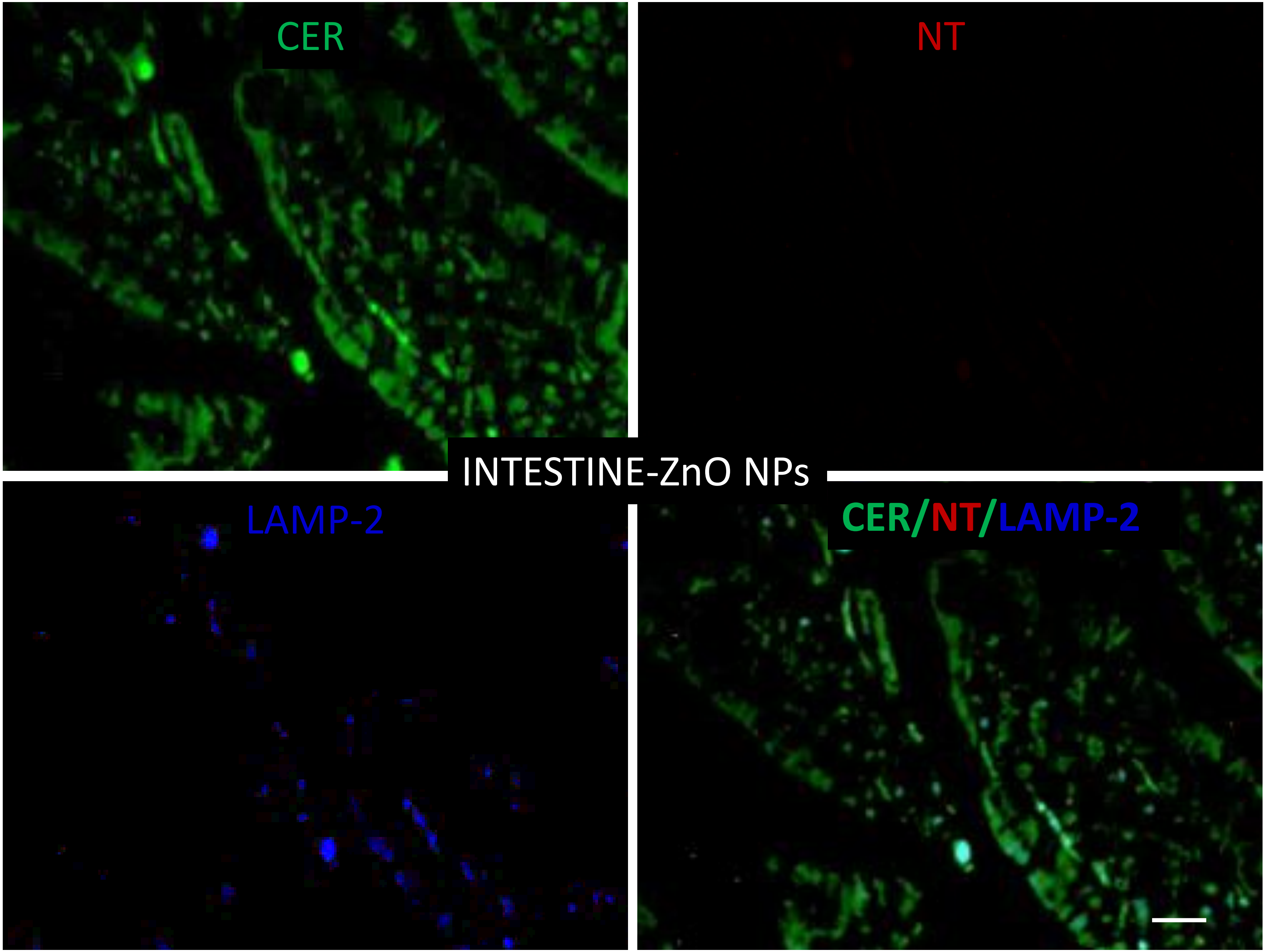
CER
NT
INTESTINE-ZnO NPs
LAMP-2
CER/NT/LAMP-2

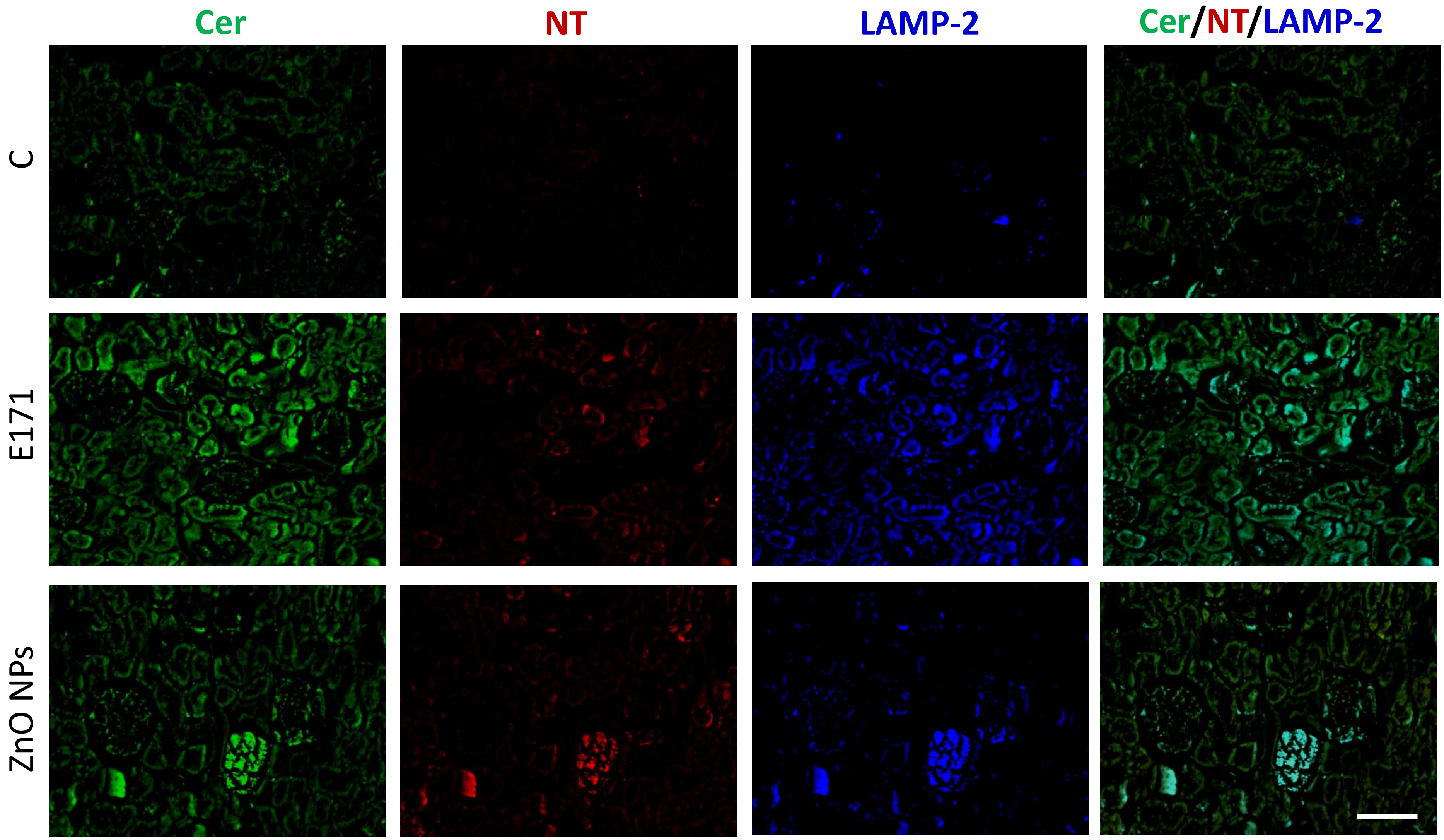


Figure S5. Representative images of ceramide (Cer), nitrotyrosine (NT) and LAMP-2 immunodetection in kidney of control (C) rats and rats treated with E171 and ZnO NPs. Bar=100μm.

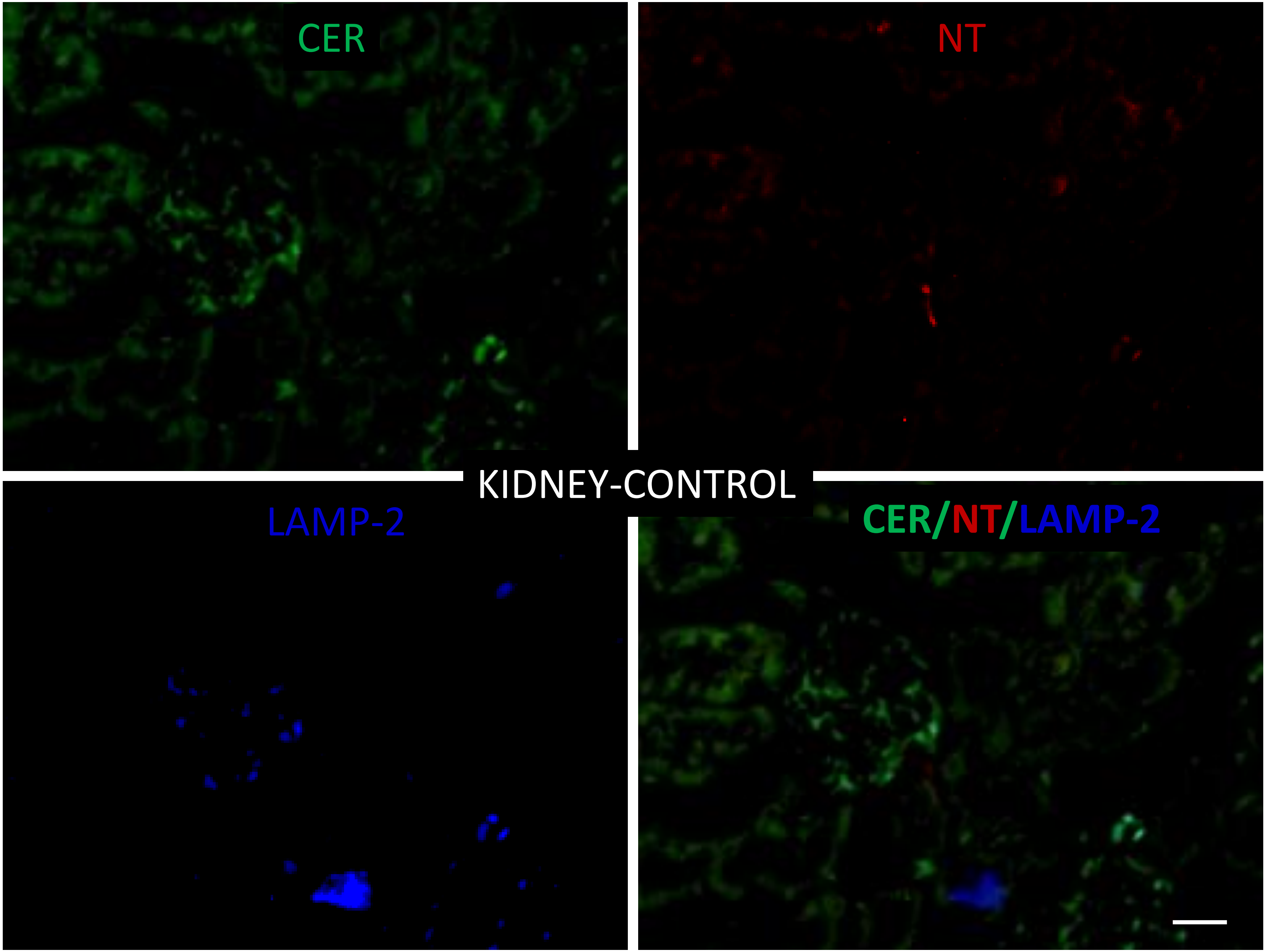
CER
NT
KIDNEY-CONTROL
LAMP-2
CER/NT/LAMP-2

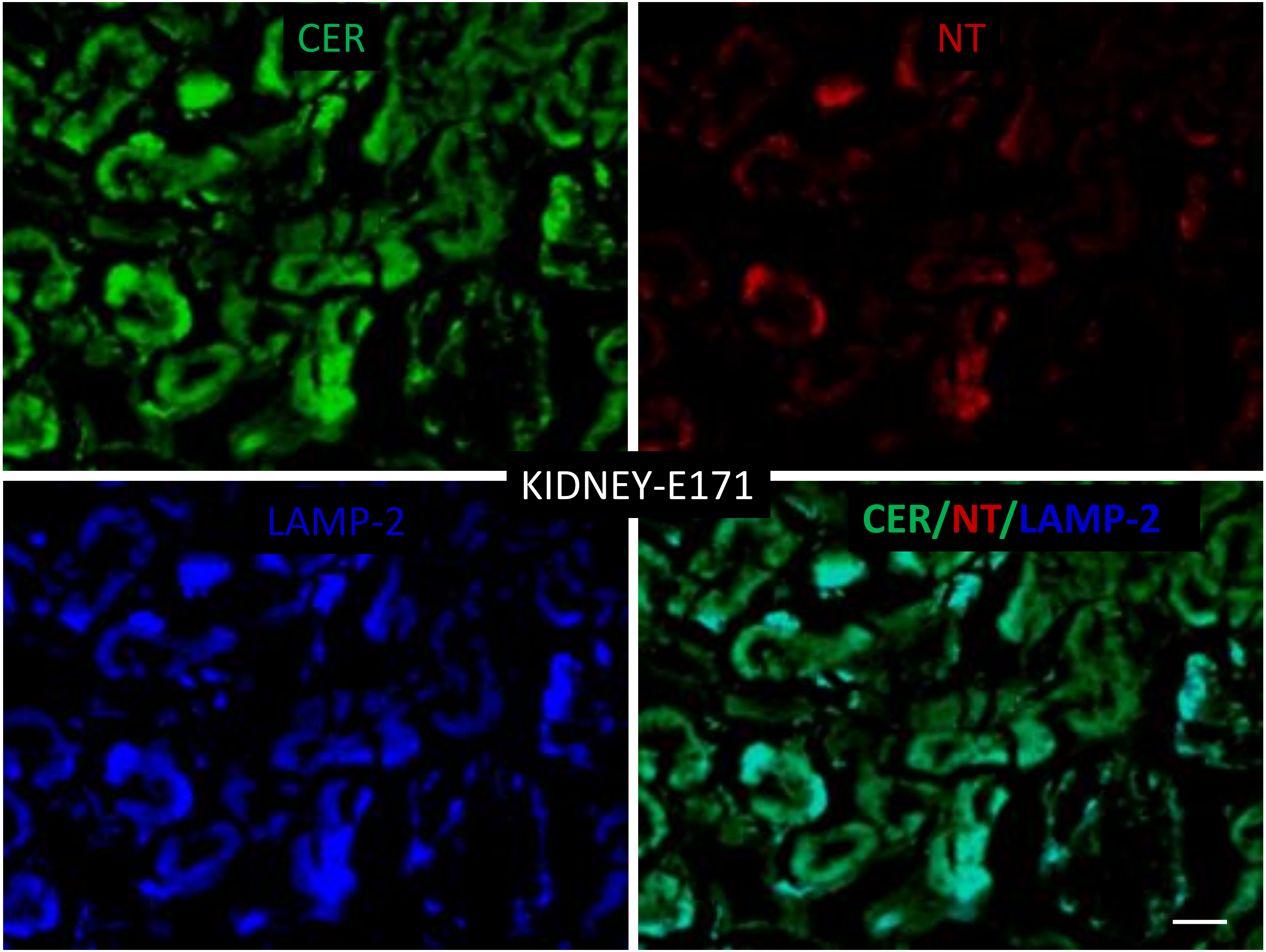
CER
NT
LAMP-2
CER/NT/LAMP-2
KIDNEY-E171

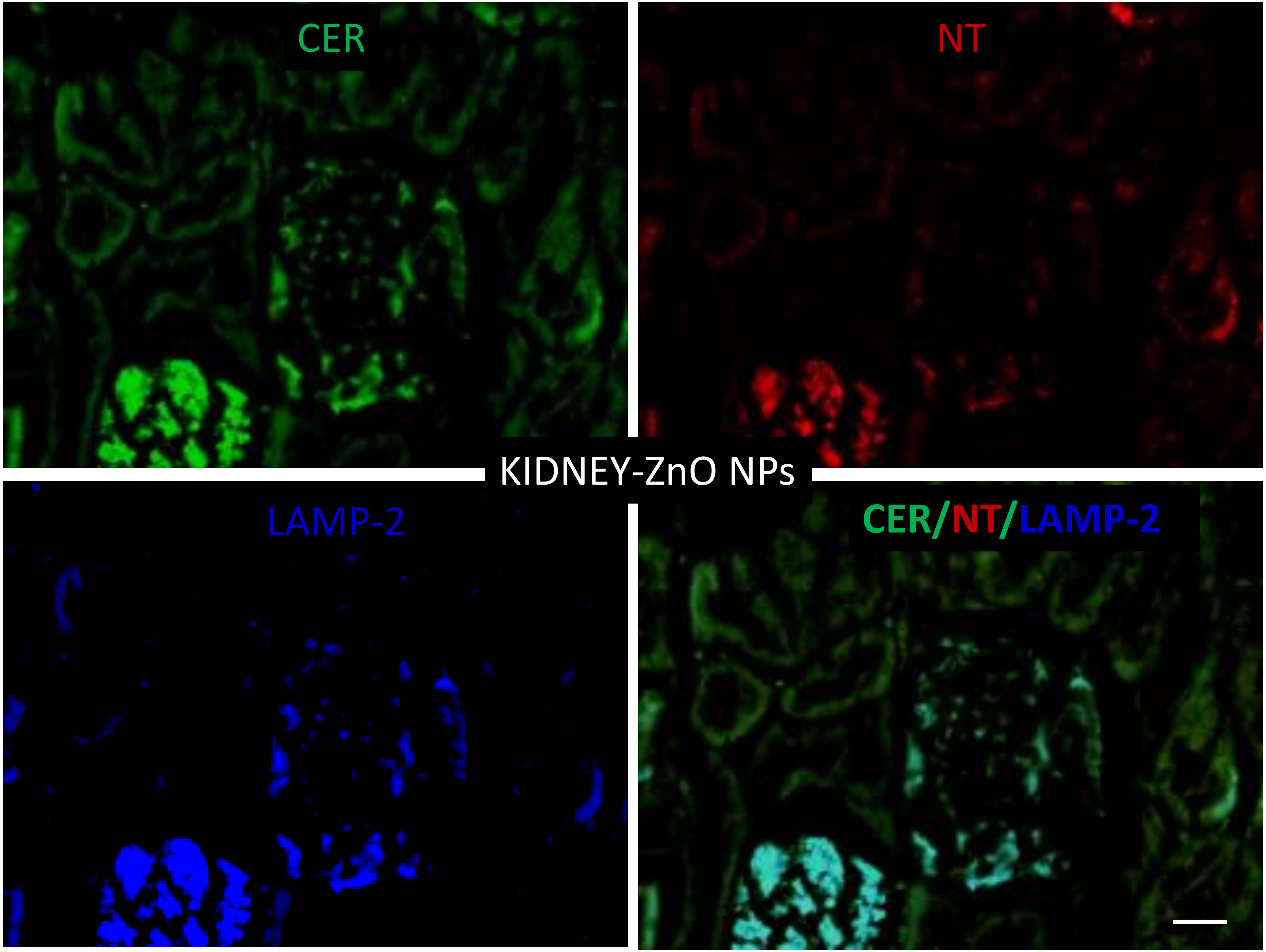
CER
NT
KIDNEY-ZnO NPs
LAMP-2
CER/NT/LAMP-2

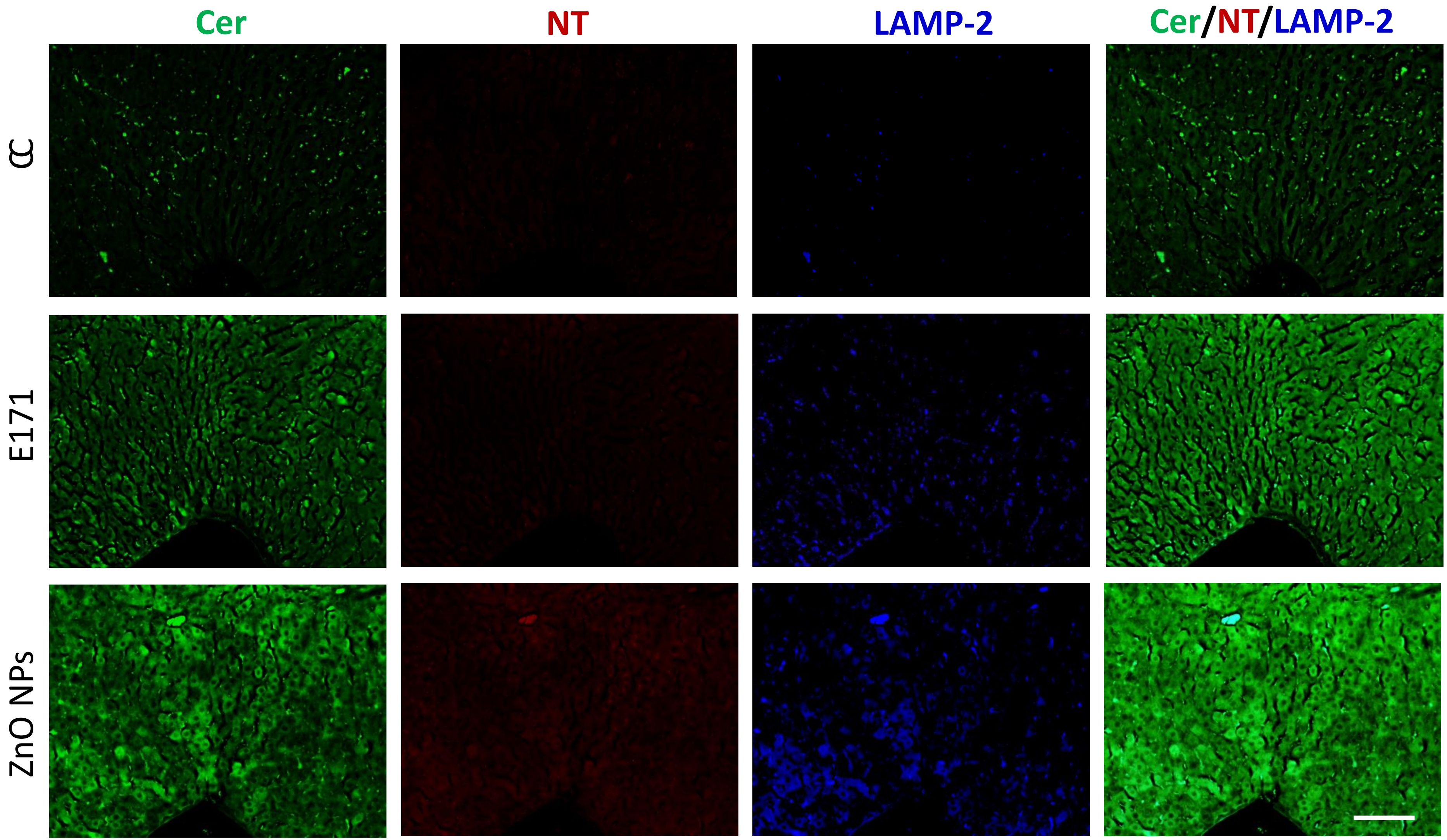

Figure S6. Representative images of ceramide (Cer), nitrotyrosine (NT) and LAMP-2 immunodetection in liver of control (C) rats and rats treated with E171 and ZnO NPs. Bar=100µm.

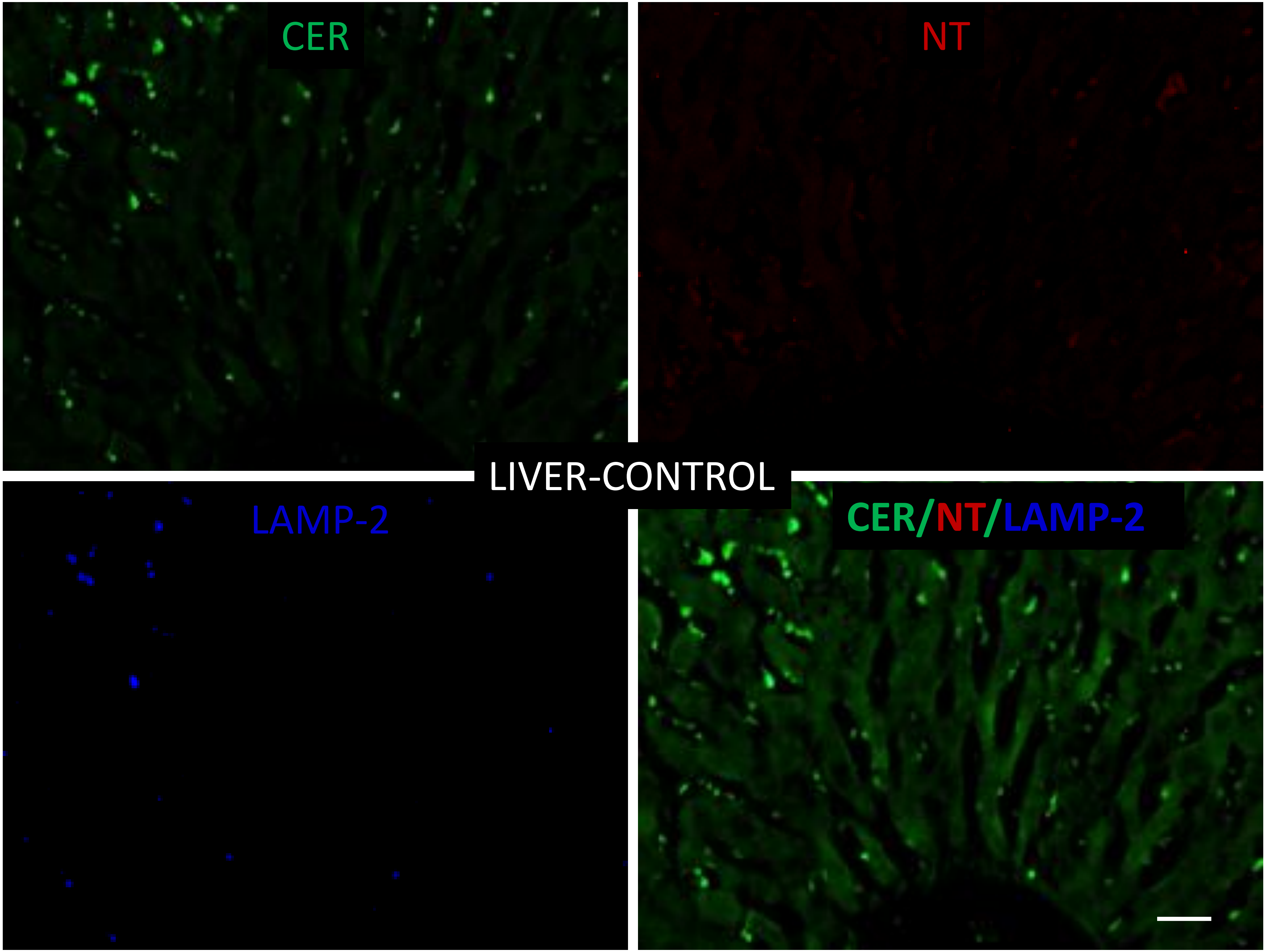
CER
NT
LIVER-CONTROL
LAMP-2
CER/NT/LAMP-2

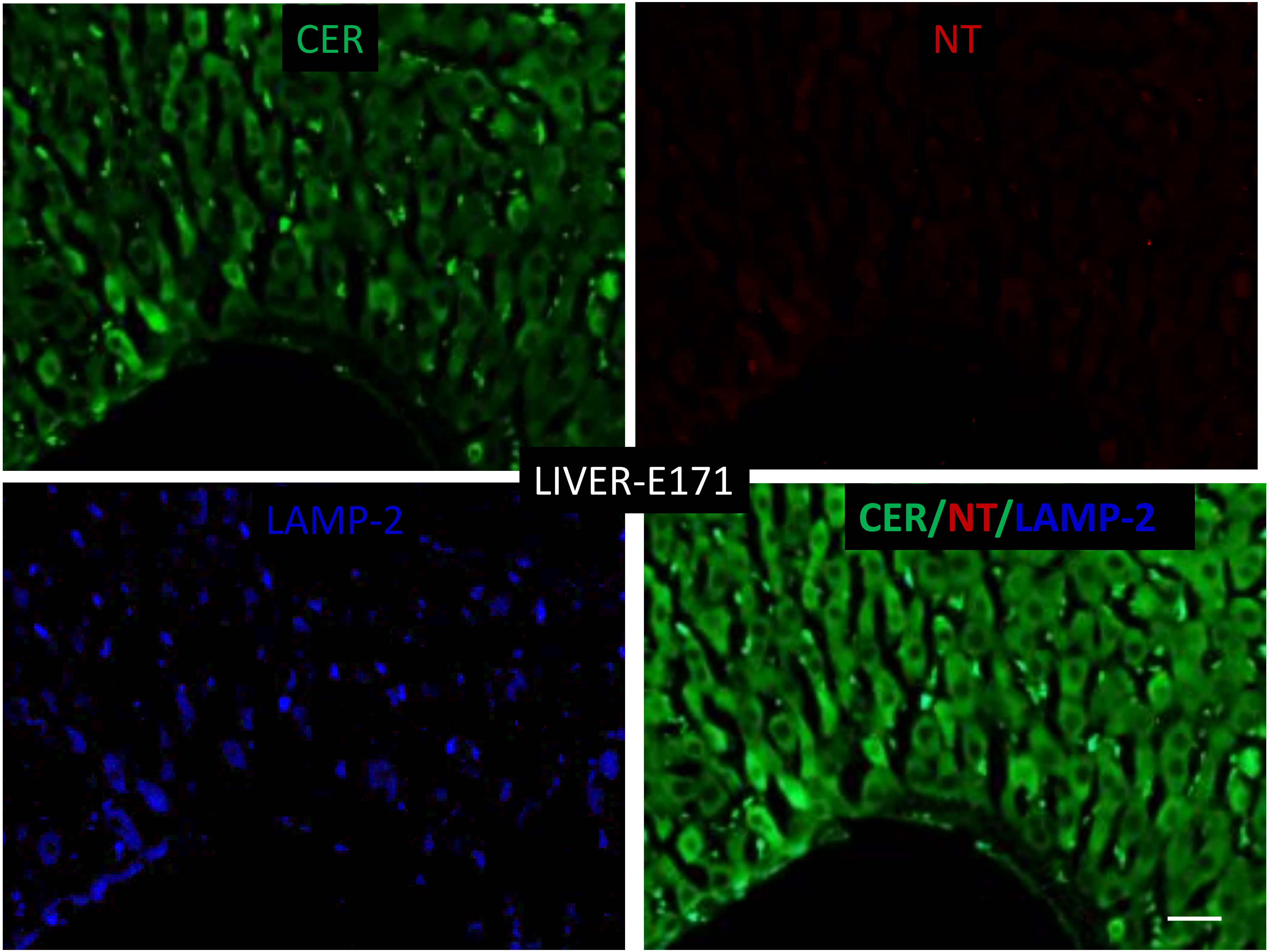
CER
NT
LIVER-E171
LAMP-2
CER/NT/LAMP-2

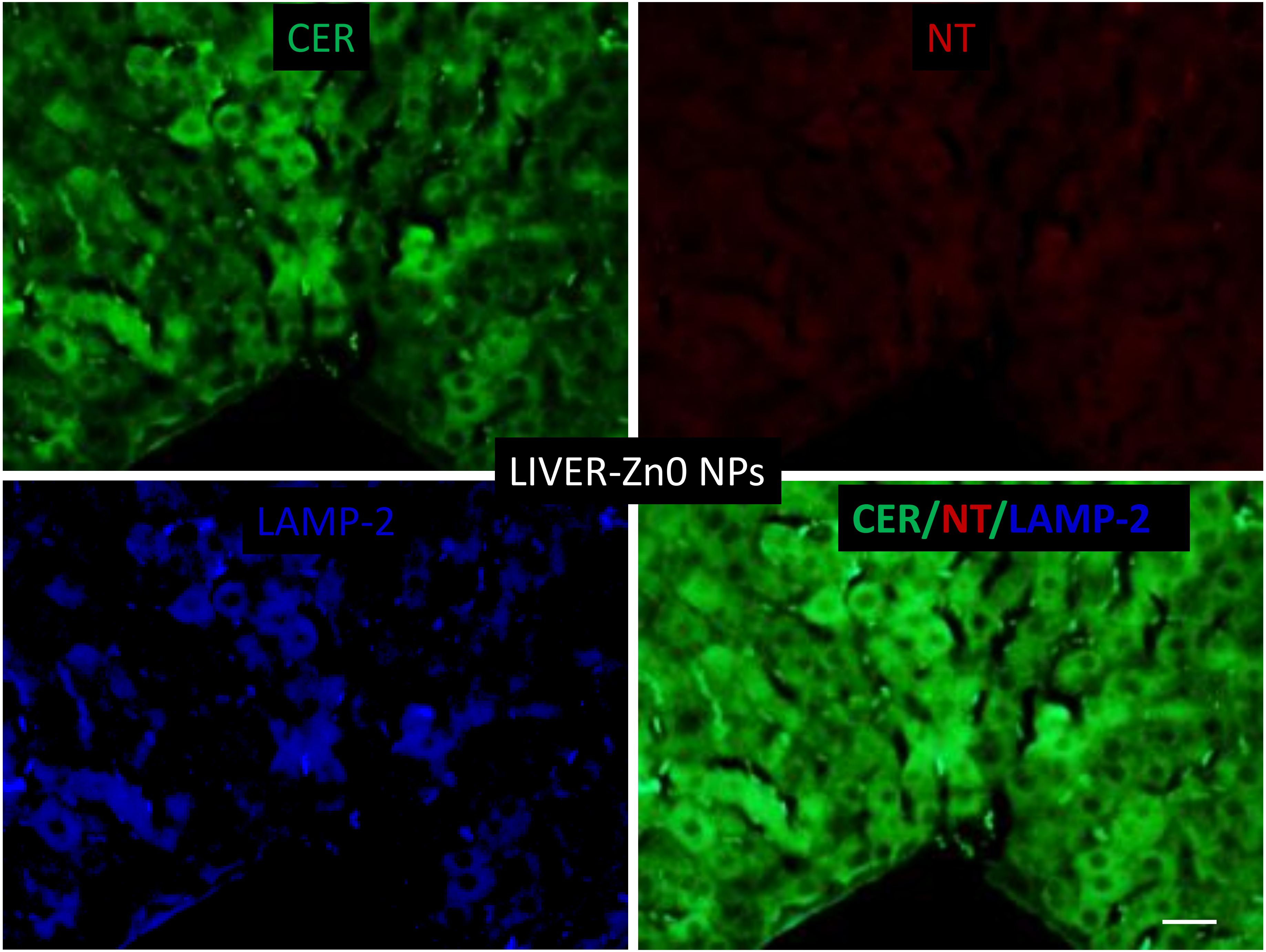

CER
NT
LIVER-Zn0 NPs
LAMP-2
CER/NT/LAMP-2
